# Semi-transparent central stop in high-resolution X-ray ptychography using Kirkpatrick–Baez focusing

**DOI:** 10.1107/S0108767313019612

**Published:** 2013-08-14

**Authors:** R. N. Wilke, M. Vassholz, T. Salditt

**Affiliations:** a University of Göttingen, Institute for X-ray Physics, Friedrich-Hund-Platz 1, 37077 Göttingen, Germany

**Keywords:** ptychography, Kirkpatrick–Baez mirrors, semi-transparent central stop, semi-transparent beam stop, coherent diffractive imaging, phase reconstruction

## Abstract

A ptychographic coherent X-ray diffractive imaging (PCDI) experiment has been carried out using 7.9 keV X-rays and Kirkpatrick–Baez focusing mirrors. By introducing a semi-transparent central stop in front of the detector the dynamic range on the detector is increased by about four orders of magnitude. The feasibility of this experimental scheme is demonstrated for PCDI applications with a resolution below 10 nm. The results are compared with reference data and an increase of resolution by a factor of two is obtained, while the deviation of the reconstructed phase map from the reference is below 1%.

## Abstract 2

A semi-transparent central stop has been used for ptychographic coherent diffractive imaging to increase the effective dynamic range in the recording of the far-field diffraction patterns. In this way, the high flux density provided by nano-focusing Kirkpatrick–Baez mirrors can be fully exploited for high resolution and quantitative phase reconstructions.

## 1. Introduction

In recent years, coherent diffractive imaging (CDI) and ptychographic coherent diffractive imaging (PCDI) have become well established high-resolution lensless X-ray imaging techniques (Miao *et al.*, 1999 ▶; Chapman & Nugent, 2010 ▶; Thibault & Elser, 2010 ▶). In contrast to lens-based X-ray microscopy they do not rely on optical elements between the sample and the detector. One or multiple oversampled diffraction patterns are recorded without the need for an imaging lens (Sayre, 1952 ▶; Miao *et al.*, 1998 ▶). The diffraction patterns are then used by iterative algorithms to reconstruct an image of the specimen (Gerchberg & Saxton, 1972 ▶; Fienup, 1978 ▶, 1982 ▶; Sayre, 1980 ▶; Bauschke *et al.*, 2002 ▶; Elser, 2003 ▶; Faulkner & Rodenburg, 2004 ▶; Thibault *et al.*, 2008 ▶; Rodriguez *et al.*, 2013 ▶). The ptychographic method which has been applied in this study has its origin in the field of electron microscopy (Hoppe, 1969*a* ▶,*b* ▶; Hoppe & Strube, 1969 ▶; Nellist *et al.*, 1995 ▶; Nellist & Rodenburg, 1998 ▶) and was introduced to the field of CDI by Rodenburg *et al.* (2007 ▶). Here the phase problem is solved by scanning a sample along directions perpendicular to the optical axis with a finite beam and a certain overlap between adjacent illuminated spots (Bunk *et al.*, 2008 ▶). The finite beam defines an effectively finite object at every scan point and the overlap between adjacent illuminated regions constrains the data. PCDI exhibits excellent convergence properties and robustness to noise (Faulkner & Rodenburg, 2005 ▶). In contrast to other CDI schemes, ptychographic algorithms are even capable of reconstructing both the possibly extended object and the illuminating wavefield (also called probe) from the same data (Maiden & Rodenburg, 2009 ▶; Thibault *et al.*, 2009 ▶). Recently, Thibault & Menzel (2013 ▶) have also demonstrated how the ptychographic scheme can be adapted to overcome effects of partial coherence and related phenomena. In order to achieve high-resolution images one has to invest sufficiently high doses (Howells *et al.*, 2009 ▶). The challenge is to simultaneously record the very intense primary beam and the quickly decaying scattering signal (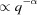, with α ≃ 4).

In view of the outlined advantages of PCDI and the dynamic range problem, it becomes obvious that PCDI in combination with focused (divergent) beams is most suitable for high-resolution imaging of extended specimens. First results using divergent beams have been reported, including nano-focusing refractive X-ray lenses (Schropp *et al.*, 2010 ▶, 2012 ▶; Hönig *et al.*, 2011 ▶), Fresnel zone plates (Vila-Comamala *et al.*, 2011 ▶; Huang *et al.*, 2012 ▶; Wilke *et al.*, 2012 ▶) and Kirkpatrick–Baez (KB) mirrors (Kewish *et al.*, 2010 ▶; Takahashi *et al.*, 2011 ▶, 2013 ▶; Huang *et al.*, 2012 ▶; Giewekemeyer *et al.*, 2013 ▶). KB mirrors are attractive focusing devices due to their high efficiency at sub-100 nm focusing while providing long working distances of hundreds of millimetres (Hignette *et al.*, 2005 ▶; Matsuyama *et al.*, 2006 ▶; Mimura *et al.*, 2007 ▶, 2010 ▶, 2011 ▶). However, efficient KB mirror optics in combination with the high coherent flux provided by modern synchrotrons such as PETRA III result in data that cannot be processed by current photon-counting detector technologies. Recently, Liu *et al.* (2013 ▶) suggested using a fully absorbing beam stop to overcome the high-dynamic-range problem. Their numerical simulations show that the missing data problem can be partly solved by PCDI. In addition, the problem has previously been approached by patching data recorded with a fully absorbing beam stop with data taken where the central part has been attenuated (Takahashi *et al.*, 2011 ▶, 2013 ▶). In this study we approached the high-dynamic-range problem by the use of a highly attenuating single semi-transparent central stop (STCS) without the need for patching two diffraction patterns and the additional recording at each scan point. We address the reliability of this scheme, give guidelines for designing the STCS and outline how it is best introduced by making use of a KB setup. We compare our STCS data with data taken without the STCS in order to validate the quantitativeness of the ptychographic reconstructions. The paper closes with an analysis about the increase in resolution and a brief discussion about future improvements in STCS fabrications.

## 2. Experiment

**Figure 1:**
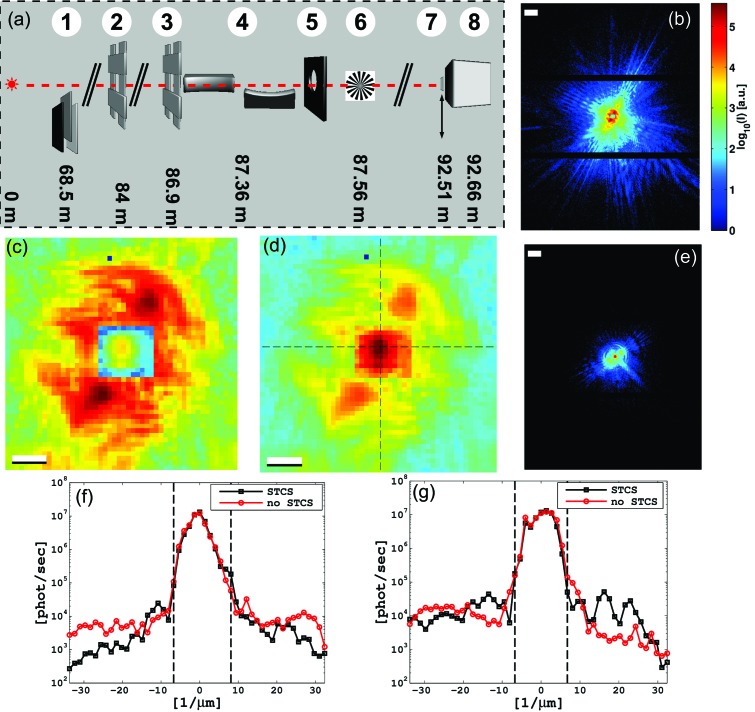
(*a*) Schematic of the setup: (1) Si attenuation foils, (2) silts S1, (3) slits S2, (4) focusing optics (KB mirrors), (5) pinhole, (6) focal region with the resolution chart sample, (7) semi-transparent central stop (STCS), (8) photon-counting detector. (*b*) Diffraction pattern taken with the STCS and 166.8 µm Si attenuation foils (1). (*c*), (*d*) Inner part from (*b*) without and with, respectively, rescaling with respect to the area attenuated by the STCS (7). (*e*) Diffraction pattern taken without an STCS but with 444.8 µm Si attenuation foils (1). (*f*), (*g*) Line profiles through the rescaled diffraction pattern shown in (*d*) [indicated by black dashed lines in (*d*)] in the horizontal and vertical direction (solid black lines), respectively. Up-scaled line profiles (scaling factor = 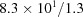 = 63.85) through a diffraction pattern taken without the STCS are shown as solid red lines. The STCS region is indicated by black dashed lines in (*f*) and (*g*). Scale bars denote 50 µm^−1^ and 10 µm^−1^ in (*b*), (*e*) and (*c*), (*d*), respectively. The exposure time is 1 s in (*b*) and (*e*). The colour bar in (*e*) is the same as in (*b*).

The ptychographic imaging experiment was carried out at the coherent nano-focus endstation GINIX (Göttingen Instrument for Nano-Imaging with X-rays) of the P10 coherence beamline of the PETRA III storage ring located at DESY, Hamburg, Germany. The photon energy was defined by a Si(111) monochromator to be 7.9 keV. The focusing setup (for a schematic see Fig. 1*a* ▶) consists of two pairs of beam-defining slits and Si attenuation foils in front of two elliptical X-ray mirrors in the KB geometry. The Pd-coated vertical mirror (WinlightX) and the horizontal mirror (JTEC) were designed for 4.05 mrad incident angle (centre) and focal distances of 302 mm and 200 mm, respectively [for details see Kalbfleisch *et al.* (2011 ▶) and Kalbfleisch (2012 ▶)]. A 5 µm-diameter W pinhole was placed at ∼1 mm in front of the KB focus in order to guarantee the oversampling condition for ptychographic phasing (Miao *et al.*, 1998 ▶) by effectively absorbing tails of the KB beam (Giewekemeyer *et al.*, 2013 ▶). In the standard mode for ptychography the sample is scanned along a rectangular grid of points in a plane of the KB focus and the resulting diffraction patterns are collected by the photon-counting pixel detector (pixel size 172 µm × 172 µm) PILATUS 300K (DECTRIS) placed about 5.1 m behind the focal plane. Absorption and background originating from air scattering are reduced by an evacuated flight tube between the sample and the detector. Note that the beam usually needs to be strongly attenuated in order to prevent damage of the detection device. The maximum count rate of the PILATUS of about 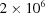
 photons s^−1^ (Toyokawa *et al.*, 2010 ▶) is exceeded by the available full coherent flux of approximately 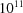
 photons s^−1^ (Salditt *et al.*, 2011 ▶). We thus expanded the setup by introducing the STCS into the flight tube at ∼15 cm in front of the photon detector [(7) in Fig. 1(*a*) ▶]. The STCS consists of a rectangular 25 µm-thick W foil (ChemPur) cut to a size of 2.2 mm × 2.0 mm by wire-cut electric discharge machining. The STCS is fixed on top of a polymide foil and can be moved in and out of the beam in front of the detector. The STCS’s theoretical transmission is 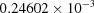 at 7.9 keV photon energy (Henke *et al.*, 1993 ▶).

From the variety of available materials that can be used for STCS applications tungsten is favoured for several reasons. Firstly, tungsten yields a high attenuation in comparison with other materials such as silicon. This is advantageous as thick layers of material are likely to produce a varying attenuation at the boundaries owing to a slight tilt with respect to the optical axis. Secondly, the main X-ray fluorescence emission lines are well above or below the energy threshold of the PILATUS 300K at 7.9 keV photon energy. Moreover, tungsten foils are commercially available in the favoured dimensions of mm × mm × µm and can be easily processed. This is particularly helpful in synchrotron applications, where the beam shape of the experiment cannot always be anticipated with an accuracy in the range of the pixel dimension of the detector before the experiment. The problem of designing the right dimensions for the STCS can thus be circumvented by fabricating a set of different sizes. Importantly, the STCS allowed us to increase the photon flux on the sample by two orders of magnitude without saturating the photon-counting device.

A first ptychographic data set (data set 1) was collected on a Siemens star X-ray resolution chart (500 nm-thick layer of tantalum with finest feature size of 50 nm; model ATN/XRESO-50HC, NTT-AT, Japan) using the W STCS in front of the detector. An important feature of the GINIX versatility arises from the slits in front of the KB optics which can be used to change the numerical aperture of the mirrors. The slits can thus be used to change the focal size, the divergence of the beam and, consequently, its imprint on the detector (Matsuyama *et al.*, 2006 ▶; Giewekemeyer *et al.*, 2013 ▶). We found an optimum match between the imprint of the beam and the area covered by the STCS by choosing a position of the slits where they slightly cut into the beam (*cf.* Table 1 ▶). At 7.9 keV the STCS attenuates the central beam by about four orders of magnitude. An additional attenuation of the X-ray beam by approximately one order of magnitude (*cf.* Table 2 ▶) was necessary to avoid saturation of the detector. From the optimum scaling factor γ of the STCS for the attenuated region of the detector, a photon flux of 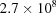
 photons s^−1^ was inferred (*cf*. Table 2 ▶). The ptychographic data consist of 441 scan points taken on a rectangular mesh with a uniform grid size of 150 nm × 150 nm and cover a scan region of 3 µm × 3 µm. The exposure time of each grid point is 1 s. The total measurement time including movements of the sample was ∼9 min.

Secondly, ptychographic data (data set 2) were collected on the same specimen without attenuating the central beam in front of the detector, necessitating a higher global attenuation of the full X-ray beam. The missing STCS attenuation was compensated by an attenuation of 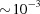 through Si attenuation foils in front of the KB optics (*cf.* Table 2 ▶). Thereby, the total photon flux available for ptychography was reduced to 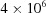
 photons s^−1^. The scanned area was 4 µm × 4 µm with a grid size of 160 nm × 160 nm. In total, 676 diffraction patterns were recorded using an exposure time of 1 s per scan point. The total measurement time including movements for scanning the sample was ∼15 min.

## 3. Analysis

The data were analysed using the ePIE algorithm (Maiden & Rodenburg, 2009 ▶). The output of the algorithm are estimations of both the complex probe 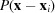 and the object 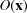 being illuminated by the probe at positions 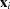. All phase reconstructions were obtained after 400 iterations as an average over the last 100 iterations and by using α = β = 0.5 (*cf.* Maiden & Rodenburg, 2009 ▶). The sample plane was initialized as uniform amplitude with constant phase, whereas the probe was initiated as a circular aperture of diameter 600 nm with constant amplitude and phase. A region of 460 × 600 pixels (h × v) around the central peak of the recorded diffraction patterns was used yielding a real-space pixel size of 10.1 nm and 7.8 nm in the horizontal and vertical directions, respectively. The insensitive horizontal stripes between adjacent detection modules of the PILATUS (*cf.* Fig. 1*b* ▶) were treated as zero-intensity regions during the iterative reconstructions.

**Figure 2:**
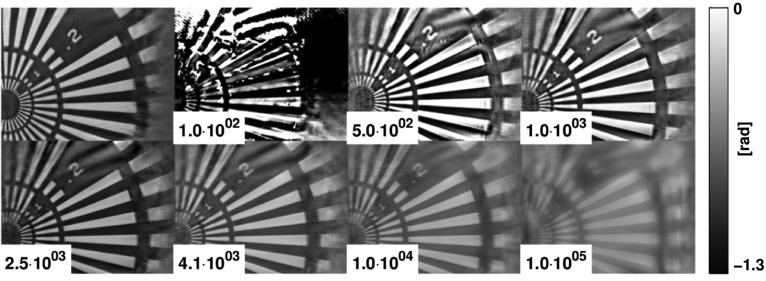
Phase reconstructions of data set 1 assuming different STCS transmission values in comparison with the phase reconstruction of data set 2 (without attenuation of the central beam) which is shown at the top left position. Corresponding scaling factors γ (1/transmission) are shown as insets.

**Figure 3:**
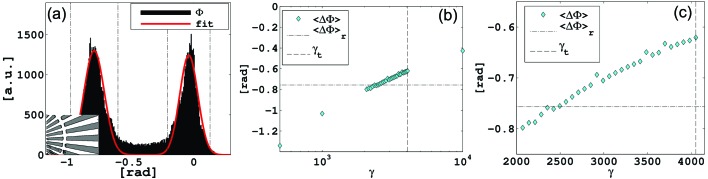
(*a*) Phase histogram corresponding to data set 2. The histogram contains only phases within the rectangular area depicted in the inset. A fit of a sum of two Gaussians to the histogram is drawn in red. The phase of Ta (or no Ta) is estimated as an average over one 2 FWHM interval (dash-dotted black lines). The intervals correspond to the white and grey regions in the graph of the inset. (*b*) Estimations of 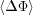 corresponding to data set 1 for different values of γ using 2 FWHM intervals of the histograms. The reference 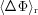 as estimated from the inset shown in (*a*) is indicated as dash-dotted black line. The theoretical value for 25 µm W is indicated as a dashed black line. (*c*) Close-up of (*b*). Here it can be seen that γ = 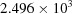 is in good agreement with the reference.

At first, the influence of different scaling factors for the attenuated central intensity of data set 1 on the reconstruction was studied. Phase reconstructions corresponding to varying scaling factors γ in the range 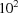 to 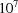 are presented in Fig. 2 ▶. The theoretical scaling factor γ = 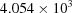 corresponding to the theoretical value of 25 µm tungsten was also included. We have compared the phase reconstructions by calculating the relative phase shift of the Siemens star resolution chart (*cf.* Fig. 3 ▶). As a criterion for reconstruction quality we evaluated the deviation of the phase from the average of the uniform regions, with and without tantalum. We used phase histograms such as can be seen in Fig. 3(*a*) ▶ to define the areas with and without Ta within a chosen rectangular region of the reconstructed phase maps by fitting a sum of two Gaussians to the histogram. We found that the binary structure of the Siemens star is well reproduced if all phases lying in a 2 FWHM (full width at half-maximum) ≃ 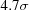 interval were included. The results of the calculated phase shifts 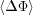 are shown in Figs. 3(*b*), 3(*c*) ▶ and Table 3 ▶.

**Figure 4:**
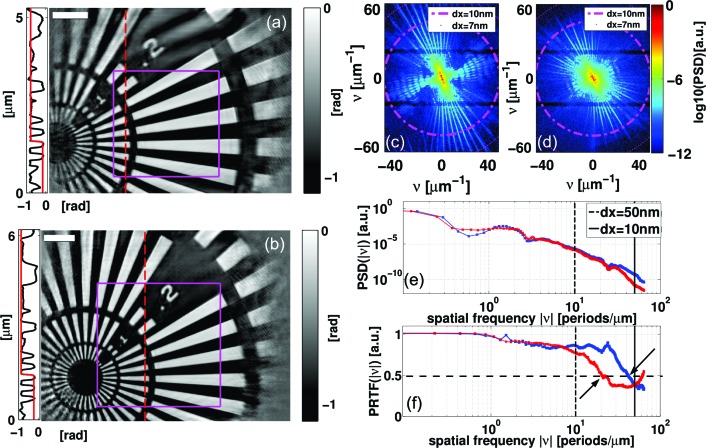
(*a*), (*b*) Phase reconstructions of the resolution chart sample as obtained from data taken with a photon flux of 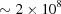
 photons s^−1^ and the STCS and with a photon flux of 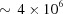
 photons s^−1^ and no STCS, data sets 1 and 2, respectively. In both cases the sample was scanned over a rectangular area as indicated by magenta lines. Fits of the error function to the edges of the line scans (dashed red lines) yield line-spread functions with FWHM values of 23 nm and 26 nm, in (*a*) and (*b*), respectively. (*c*), (*d*) Calculated two-dimensional power spectral densities (PSDs) of the reconstructed phases depicted in (*a*) and (*b*), respectively. Frequencies corresponding to a real-space pixel size of 10 nm and 7 nm are indicated as magenta circles. (*e*), (*f*) PSDs and phase retrieval transfer functions (PRTFs) from both data sets after azimuthal averaging. Data set 1 is represented in blue whereas data set 2 is represented in red. The arrows in (*f*) indicate the PRTF cutoff at about 0.5 yielding a half-period resolution of 12 nm and 25 nm for data sets 1 and 2, respectively. Frequencies corresponding to real-space pixel sizes of 50 nm and 10 nm are further emphasized in (*e*) and (*f*) by vertical lines. Scale bars denote 1 µm in (*a*) and (*b*).

**Figure 5:**
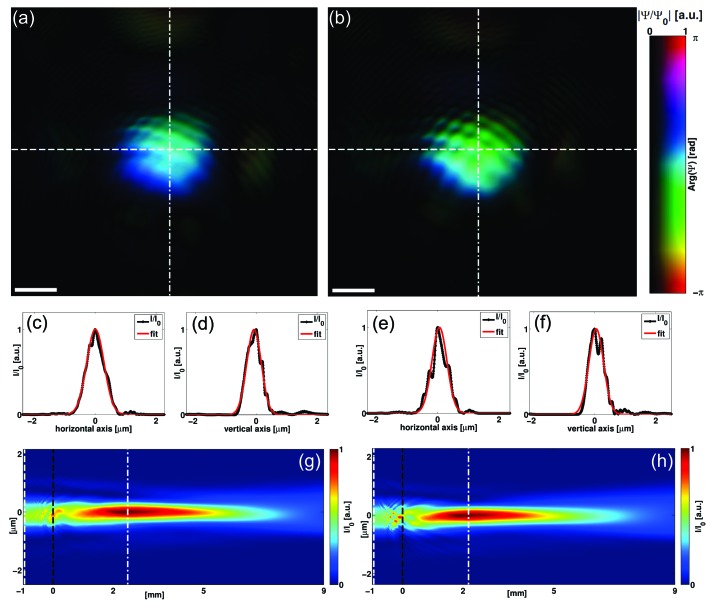
(*a*), (*b*) Visualizations of the central part of the reconstructed complex probe fields of data set 1 and data set 2, respectively. (*c*) Intensity of a horizontal slice through the probe at the sample [dashed white line in (*a*)]. (*d*) Intensity of a vertical slice through the probe at the sample [dash-dotted white line in (*a*)]. Gaussian fits (red lines) yield FWHM values of 673 nm × 564 nm (h × v). (*e*) Intensity of a horizontal slice through the probe at the sample [dashed white line in (*b*)]. (*f*) Intensity of a vertical slice through the probe at the sample [dash-dotted white line in (*b*)]. Gaussian fits (red lines) yield FWHM values of 580 nm × 566 nm (h × v). (*g*), (*h*) Intensity of numerically propagated probe fields as a slice through the optical axis and the horizontal axis [indicated as a dashed white line in (*a*), (*b*)] of the focus (at 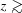
2 mm) of data sets 1 and 2, respectively. Dashed white lines, dashed black lines and dash-dotted white lines indicate the plane of the pinhole, position of the sample and position of the focal plane, respectively. Scale bars denote 0.5 µm in (*a*) and (*b*).

Finally, we present a quantification of the achieved resolution on the reconstructed phase maps of the reference data set 2 and the STCS data set 1 where the scaling factor γ = 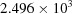 yields the best agreement with the reference. Reconstructed phase maps corresponding to data sets 1 and 2, which are already shown in Fig. 2 ▶, are presented in Figs. 4(*a*) and 4(*b*) ▶, respectively. The reconstructed probe fields are presented in Figs. 5(*a*) and 5(*b*) ▶. We analysed the power spectral density (PSD) of both reconstructed phase maps. The two-dimensional PSDs were calculated by using a Kaiser window (*cf.* Wilke *et al.*, 2012 ▶; Giewekemeyer, 2011 ▶) and are presented in Figs. 4(*c*) and 4(*d*) ▶. In order to better compare the frequency content of the reconstructions, the two-dimensional PSDs have been azimuthally averaged and are depicted in Fig. 4(*e*) ▶.

Although the PSD gives a proper measure of the frequency content of the reconstructed phase map, the resolution achieved by ptychographic phasing needs to be carefully justified. For this reason we have also calculated the phase retrieval transfer function (PRTF) which compares the measured amplitudes 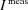 with the reconstructed ones 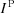 (*cf.* Shapiro *et al.*, 2005 ▶; Chapman *et al.*, 2006 ▶; Giewekemeyer *et al.*, 2010 ▶). As discussed by Wilke *et al.* (2012 ▶), the PRTF can be adapted to the scanning nature of ptychography,
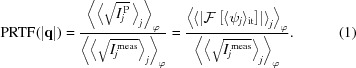
Here, 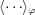, 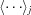, 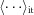 denote azimuthal average, average over scan positions and average over the last iterations, respectively. The PRTFs of both data sets are shown in Fig. 4(*f*) ▶.

## 4. Results and discussion

A comparison of line profiles through the centre of diffraction patterns from data sets 1 and 2 [Figs. 1(*f*) and 1(*g*) ▶] shows a good agreement of the rescaling in the centre region of the diffraction pattern of data set 1 with the one corresponding to data set 2.

Next, we have analysed the influence of different scaling factors γ for the STCS region of data set 1 (*cf.* Fig. 2 ▶). By comparison with the reconstruction obtained without the STCS (data set 2), one can read off an optimum scaling factor of 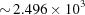, which deviates by a factor of 0.62 from the theoretical value. The comparably large relative uncertainty 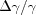 = 0.62 can be explained by the Beer–Lambert law. Applying the Beer–Lambert law yields the dependence of the relative uncertainty of the thickness of the used STCS foil 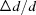 on the relative uncertainty of the scaling factor: 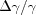 = 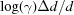. The explicit occurrence of the scaling factor implies an accurate knowledge of the STCS thickness. For instance, using 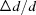 = 0.1 and γ = 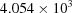, which correspond to the experimental parameters, yields 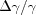 = 0.83, which is in good agreement with the experimental result.

For the optimum scaling factor γ ≃ 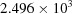 the reconstructed phase shift of 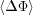 = −0.755 rad (Ta *versus* no Ta) deviates by less than 1% from the reference value (*cf.* Table 3 ▶). Interestingly, a lower than optimum scaling factor results in an increased phase contrast of the object. The tolerance for the reconstruction is surprisingly large, spanning roughly one order of magnitude between the lowest tolerable value and the optimum. Towards higher values than optimum, the tolerance is even larger; although losing more and more phase contrast, the object can still be clearly identified at an over-scaling of three orders of magnitude. The reason for this result may be explained as follows: assuming that the STCS region contains only low-frequency information about the probe, the intensity scaling acts as a filter that shifts the relative contributions between object and probe. If the scaling is too low (in the limit of becoming a fully absorbing beam stop) the information about the probe is highly suppressed in the data causing a violation of the probe assumptions in the algorithm. With an increasing scaling factor γ, the frequency contributions from the object become more suppressed.

The visual impression of the high-resolution phase maps [Figs. 4(*a*) and 4(*b*) ▶] is very good. The innermost Siemens star ring structure ending at a width of 50 nm can be clearly resolved by both data sets. In the case of data set 1 this is already a hint for the high resolution obtained as the innermost structure does not fall into the scanned area marked by a magenta rectangle. The part outside this area is only illuminated by the tails of the illuminating wavefield and is thus illuminated by considerably fewer photons. The resolution can be further analysed by fitting an error function to one of the edges occurring in the image. We obtained line-spread functions with an FWHM of 23 nm and 26 nm at singular positions in data set 1 and data set 2 [Figs. 4(*a*) and 4(*b*) ▶], respectively. The calculated two-dimensional PSDs yield a resolution estimation of the whole phase maps [Figs. 4(*c*) and 4(*d*) ▶]. Note that there are two horizontal stripes in the two-dimensional PSDs which contain less information about the sample due to the insensitive regions of the detector between the modules. Besides, one can clearly see the frequency contributions arising from the star form of the object. We find frequency contributions corresponding to pixel sizes in the range from 10 nm down to 7 nm for data set 1, whereas the frequency content of data set 2 seems to end at 10 nm. The azimuthal average of the PSDs as shown in Fig. 4(*e*) ▶ confirms our result. In the high-frequency part of the spectrum the curve of data set 1 (blue curve) lies well above that of data set 2 (red curve). The difference in resolution is seen more drastically by comparing the PRTFs (Fig. 4*f* ▶). Now the resolution is judged by the frequency where the PRTF drops below the cutoff of 0.5. We obtain a half-period cutoff corresponding to a pixel size of 12 nm and 25 nm for data sets 1 and 2, respectively. In our opinion, the cutoff yields an upper limit and the actual resolution is better.

Next, we address the question of how well the probe of the rescaled STCS data can be recovered. We thus present the probes as reconstructed in the plane of the sample in Figs. 5(*a*) and 5(*b*) ▶. We find that the probe reconstruction from the STCS data is in good agreement with the probe reconstruction without the STCS (reference). Both probes exhibit a similar shape and fringe pattern which arises from a non-optimum alignment of the pinhole. The small differences in the fringe pattern are likely to be due to a drift of the pinhole in the time between recording data sets 1 and 2. The reliability of the reconstruction is further supported by the similarity of the propagated probe fields along the optical axis [Figs. 5(*g*) and 5(*h*) ▶]. Here we point out again that the comparably large probe size with FWHM values of 673 nm × 564 nm [h × v; data set 1; Figs. 5(*c*) and 5(*d*) ▶] and 580 nm × 566 nm [h × v; data set 2; Figs. 5(*e*) and 5(*f*) ▶] arises from the particularly small slit setting, which has been chosen to match the beam size on the detector to the size of the STCS. The quality of the obtained reconstructions shows that this beam size does not represent a limitation. On the contrary, the high resolution and relatively large beam are an ideal combination in view of imaging a large field of view and tomography.

## 5. Summary and conclusion

In summary, we have performed a study on the use of an STCS for hard X-ray ptychographic imaging. By using the STCS, the central beam on the detector is attenuated by about four orders of magnitude and thereby the usable photon flux for ptychography could be increased by two orders of magnitude. The fact that the usable flux could not be increased further, for example by further reduction of the global attenuation factor, was due to the specific nature of the test pattern. It turns out that low-frequency components contribute to a high scattering intensity very close to the central beam [*cf.* Figs. 1(*c*) and 1(*d*) ▶]. Hence, the efficiency of the STCS also depends on the object being imaged. For instance, a biological specimen with uniform scattering distribution may be studied with a lower global beam attenuation.

We have compared the STCS data with reference data taken without the STCS and found that the phase deviation for optimum scaling factors between both reconstructions is less than 1%. We would like to stress the point that usual error specifications for tungsten foils of 25 µm thickness are in the 10% range, which already has a great impact on the actual transmission. Hence, one may be tempted to invest more into the quality of the material used for STCS application. This might not be necessary for all applications because a relaxation of the requirement on quantitativeness of the phase of the object relaxes the necessary absolute value used for rescaling of the attenuated region on the detector.

We found that the reconstruction process is quite robust when changing the scaling of the STCS region by multiple orders of magnitude. In addition, we estimated the increase of resolution to be up to a factor of two, depending on the criterion used, when using the STCS. The resolution achieved is 12 nm over large parts of the sample and may be as good as 7 nm in particular regions.

The STCS technique at the Göttingen Instrument for Nano-Imaging with X-rays can still be improved, as the optimum fully available coherent photon flux of 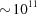
 photons s^−1^ is still almost three orders of magnitude away from the current experimental setting. In future, we will thus study the feasibility of higher attenuation factors as well as STCSs with compound attenuation values, *i.e.* a more complex attenuation profile.

An important future application may arise from ptychographic imaging of biological specimens or other weakly scattering objects. The STCS application is not restricted to ptychographic imaging. In CDI applications the central beam is typically fully blocked by a suitably dimensioned central stop and the desired image can still be reconstructed. The introduction of an STCS in CDI may relax the demands on choosing the right dimensions while simultaneously providing access to the low-*q* information about the direct beam without any change of the setup. For instance, a slight change of the beam centre could be observed while recording data. Furthermore, the STCS will find useful application in classical diffraction analysis, where one also loses low-*q* information through the use of a fully absorbing beam stop.

## Figures and Tables

**Table 1:** Information on the slit settings used

Slits	Horizontal gap (µm)	Vertical gap (µm)
S1	40	100
S2	100	100

**Table 2:** Information on Si attenuation foils used and attenuation with respect to the W STCS in front of the detector; the measured photon flux on the sample is also shown

Data set	Transmission of Si foils	Transmission of W STCS	Photon flux (photons s^−1^)
1	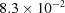	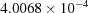	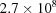
2	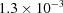	1	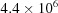

**Table 3:** Average phase shift (Ta *versus* no Ta) for the Siemens star test pattern as obtained through ptychographic phasing For the case of using an STCS, the dependence of different STCS scaling factors on the phase shift 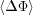 is listed.

Data set	STCS scaling factor γ	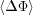 (rad)
2	1	−0.757
1	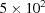	−1.341
1	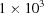	−1.032
1	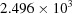	−0.755
1	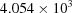	−0.621
